# Digital economy development boosts urban resilience—evidence from China

**DOI:** 10.1038/s41598-024-52191-4

**Published:** 2024-02-05

**Authors:** Haohui Wang, Gang Peng, Hongmei Du

**Affiliations:** 1 School of Statistics, Southwestern University of Finance and Economic, Chengdu, Sichuan China; 2 School of Public Administration, China University of Geosciences, Wuhan, Wuhan, Hubei China

**Keywords:** Sustainability, Socioeconomic scenarios

## Abstract

Focusing on the impact of the digital economy on urban resilience is beneficial to the sustainable development of cities. This paper empirically examines the impact of digital economic development on urban resilience and its mechanisms by measuring urban resilience and the level of urban digital economy with the entropy-weighted TOPSIS method using the data of 252 Chinese cities from 2011 to 2020. The findings show that digital economic development effectively promotes urban resilience at the 1% significance level, and this conclusion remains valid after a series of endogeneity and robustness tests. The channel mechanism suggests that the development of the digital economy can improve urban resilience by optimizing urban distributional effects and promoting the upgrading of urban industrial structures. This paper discusses the nonlinear relationship between the two using the MMQR model and the threshold model. The results show that urban resilience development level is in a higher quartile of cities, and digital economy development has a greater impact on urban resilience improvement. Meanwhile, there are two threshold values for the nonlinear impact of the digital economy on urban resilience, which are 0.026 and 0.082, respectively. Further, the spatial effect between the two is also verified. From the perspective of heterogeneity analysis, the digital economy development of high-class cities, key city clusters, and cities in eastern and western regions has a greater effect on urban resilience. This study can provide ideas and inspiration for countries to enhance urban resilience and promote sustainable urban development through the development of the digital economy.

## Introduction

In recent years, sustainable development has become an important direction for the future development of cities due to various negative impacts such as climate change, natural disasters, and resource crises^1^. The 17 Sustainable Development Goals (SDGs) issued by the United Nations also emphasize the need for inclusive, safe, risk-resistant, and sustainable cities and human settlements. In order to achieve this goal, countries have proposed a variety of highly competitive urban development strategies, such as sustainable cities, smart cities, urban resilience, green cities, etc.^2,3^. With the outbreak of the COVID-19 coronavirus, the term urban resilience (Ur) once again stood out among the many urban development pathways as an important concept for assessing sustainable urban development^4^. Ur is mainly defined as the ability of a city to resist or adapt to some external disturbance and return to normal functioning, which is a reflection of the level of urban integration^5^.

Existing literature related to urban resilience focuses on four main areas: socio-economic and cultural, local governance, overview studies, and disaster studies^6^. Among them, socio-economic and cultural-related literature is the most voluminous, focusing on the synergistic relationship between socio-economic domains such as economy, ecology, culture, resources and infrastructure, and urban resilience^7^. Local governance-related literature mainly argues for the feasibility and effectiveness of specific urban resilience public policies or administrative decisions used to support public policies^8^. In addition, a large body of literature has conducted overview studies on Ur-related topics such as rural town resilience, disaster resilience, climate change resilience, and infrastructure resilience, emphasizing that the scope of urban resilience research should be broadened and increased^9^. Finally, disaster research links urban resilience to natural hazards such as floods, climate change, earthquakes, droughts, and hurricanes and explores how cities can increase urban resilience to resist exogenous hazards^10^.

At the same time, the rapid development of the digital economy (Dig) has also affected all aspects of the city. The advent of multiple disasters has gradually made people realize that the traditional urban development model has many limitations, such as slow speed and low efficiency^11^. On the contrary, digital development, based on its efficiency and convenience, has gradually become an important path to guide cities towards sustainable development^12,13^. In this regard, the United Nations has also declared that digital technology-driven urban development is necessary to achieve the 2030 Sustainable Development Goals^14,15^. Therefore, discussing the relationship between Dig and Ur is important for urban sustainability research.

Existing research on Dig and Ur focuses on the impact of Dig on economic resilience, ecological resilience, social resilience, and infrastructure resilience. Among them, a large amount of literature focuses on the relationship between Dig and economic resilience. For example, part of the literature suggests that both the breadth of coverage and depth of use of Dig have a large positive impact on the economic resilience of cities and that innovative technology and consumption level are two important mediating variables^16^. Some of the literature suggests that Dig not only promotes regional economic resilience but also has positive spatial spillover effects^17^. Some of the literature also suggests that digital transformation can effectively improve energy efficiency, which in turn improves economic resilience^18^. In addition, some literature has also investigated the impact of Dig on urban ecological resilience. For example, part of the literature discusses the role of digitization in resisting climate change, natural disasters, etc., and proposes that digital information research and information technology can effectively enhance the ability of cities to resist environmental risks^19,20^. On the relationship between digitization and urban social resilience and infrastructure resilience. Part of the literature proposes that the development of Dig has a potential impact on the healthcare system, which can significantly increase the resistance and resilience of cities to external shocks^21^. Part of the literature suggests that Dig is an important factor in promoting electricity intensity in cities^22^.

The combination reveals the following possible shortcomings of the existing studies: first, most of the literature has explored economic resilience, social resilience, ecological resilience, and infrastructure resilience, respectively, and among them, economic resilience research is predominant, and very few of them have systematically investigated how the Dig affects the Ur. Second, most of the literature on the Ur is relatively simple in construction, such as the common resilience construction index (RCI), which often uses ten or so indicators to determine the level of regional resilience. Because the concept of resilience covers a wide range, fewer indicators make it difficult to ensure the comprehensiveness of the analysis^23,24^. Third, most Ur studies emphasize time evolution and linear relationships, while nonlinear and spatial effects are rarely explored.

The contribution of this paper may be reflected in the following aspects: First, it adds a new research perspective. Existing studies pay less attention to the impact of Dig on the overall resilience of cities, and most of them only analyze the economic resilience or ecological resilience of cities. Second, it is innovative in the construction of indicators. In this paper, Ur is measured in detail from four aspects: 11 dimensions and 23 refined indicators. Dig is measured in depth using two dimensions and five refined indicators. Third, it provides a comprehensive and rigorous argumentation idea. On the one hand, this paper adopts alternative indicators, lagged variables, instrumental variables, and other methods to explore the linear relationship between the two. On the other hand, this paper introduces the MMQR model, threshold, and spatial measure to explore the nonlinear relationship between the two. At the same time, this paper analyzes the heterogeneity based on city classes, city clusters, and regions, trying to explore the relationship between the two in a more detailed and comprehensive way.

## Theoretical mechanisms and hypothesis development

Dig is a new kind of economic form that has all-factor digital transformation at its core^25^. Ur refers to the ability of a city to withstand or adapt to certain external disturbances and return to normal functioning, and it is a reflection of the level of urban integration^5^. Dig affects Ur in four main ways: economic, social, ecological, and infrastructural. Firstly, from an economic point of view, digital technology's utilization can effectively reduce production costs, improve production efficiency, promote economic development, and enhance urban economic resilience. At the same time, the establishment of digital economic platforms improves the convenience of economic and financial activities, breaks through the traditional transaction barriers, and promotes the sustainability of economic development, which in turn enhances the city's economic resilience^26^. Second, Dig also has multiple impacts on social development. On the one hand, based on permeability and low marginal costs, Dig can reduce geographic constraints, promote the sinking of education and healthcare, and improve social security and stability^27,28^. On the other hand, Dig can also provide residents with more employment opportunities and a favorable entrepreneurial environment, adjust the income distribution in society, narrow the income gap, and thus enhance the social resilience of cities^29^. Thirdly, Dig can reduce production energy consumption, reduce pollution emissions, and promote ecological sustainability through system intelligence. In addition, based on big data analysis and digital devices, real-time monitoring of the ecological environment can be realized, reducing the occurrence of pollution incidents and effectively enhancing urban ecological resilience^30^. Fourth, the development of Dig has led to the intelligent upgrading of traditional infrastructures and facilitated the construction of high-tech infrastructures, such as information networks, to enhance urban infrastructure^1^.

### H1:

Dig can effectively contribute to Ur.

The development of Dig optimizes the urban distribution effect. Dig is centered on a new generation of digital technologies such as cloud computing and big data, and the deep integration of these technologies improves the efficiency of urban resource allocation and contributes to the realization of an individual's own value. At the same time, advanced technology provides opportunities for multiple interests to participate in the distribution, reduces the cost for each subject to participate in the distribution of interests, and is more conducive to the realization of the distribution of fairness and the realization of a win–win situation for multiple subjects. And the optimization of the distribution effect helps cities enhance Ur. On the one hand, fair and friendly distribution helps to improve the wage level of residents in the city, realize ideal income distribution, and ultimately form a strong population aggregation effect to provide reserve power for the city's innovative development. On the other hand, a good distribution effect can promote urban economic growth and reduce urban poverty rates, enhancing the ability to achieve stable social development^31^.

### H2:

Dig can enhance Ur by optimizing distributional effects.

First, Dig has a positive effect on industrial structure upgrades. On the one hand, digital technology can help traditional industries realize industrial digitalization. Traditional industries can make use of digital technology to accelerate the transformation of production factors, which in turn creates a new logic of value generation and distribution through the updating of products or business models and promotes the optimization and upgrading of the industry^32^. On the other hand, the growing Dig has brought about digital industrialization. The development of Dig has broadened the technological boundaries, promoted the restructuring and differentiation of industries, and formed human-technology-based emerging industries centered on data and information technology, further promoting the upgrading of the industrial structure^33^.

Meanwhile, the upgrading of industrial structures has a greater positive impact on enhancing the city's ability to resist and adapt to external shocks. On the one hand, industrial diversification can directly diversify the risk of external shocks and reduce the vulnerability of urban systems. On the other hand, according to Marshall's theory of externality and Jacobs' theory of externality, the upgrading of industrial structure and agglomeration can help industrial innovation, and innovative technology and knowledge have externality in the agglomeration area, which can promote the development of the city in the direction of knowledge-intensive and technological specialization^34^. Innovative and advanced technologies can effectively enhance the resistance of cities to external shocks and improve Ur^35^.

### H3:

Dig can improve Ur by promoting industrial structure upgrades.

Dig itself shows an exponential growth trend, and its impact on Ur is bound to be more than just linear. At the same time, the core of Dig is digital technology, and as with other innovations, the growth and adoption of digital technology continue to follow the technology adoption life cycle, i.e., there are innovators, early adopters, early majority, late majority, and laggards in the diffusion and adoption of digital technology. Based on this, the extent and intensity of the impact of Dig on Ur depends largely on the rate of adoption of digital technology, the extent of digital infrastructure, and the extent to which digital technology enhances the utilization of resources^36^.

In the early stages of the development of Dig, the construction of digital infrastructure and equipment requires more input from production factors. At this time, the benefits generated by the technology spillover effect are not enough to cover the factors of production consumed in its development. Therefore, with the continuous input and consumption of resources, the city's ability to resist external changes will gradually decrease and face greater development risks^37^. In the mature stage of Dig, cities can realize high-efficiency production and information breakthroughs through technological means and realize the expansion of Dig scale at a lower marginal cost. At the same time, the integration of digital technology improves the overall resource allocation level of the city, optimizes the city's energy supply structure, and strengthens the ability to cope with systemic risks. Furthermore, cloud computing, big data, and other cutting-edge digital technologies have a greater advantage in risk prediction and real-time monitoring, which can help cities do a good job of risk management and ultimately enhance the resilience of the city^38^.

### H4:

The development of Dig has a nonlinear spillover effect on improving Ur.

There is no unified view on how the development of Dig in cities affects the surrounding cities.

On the one hand, the development of Dig may create a digital divide and bring about uneven development^39^. According to the Matthew effect, due to different initial endowments, the development of Dig-related infrastructure and technology levels varies. The higher the concentration of digital economy platforms and technologies, the greater the competitive advantage of the city, and the surrounding high-quality factors of production are more concentrated in the city^40^. In contrast, it is more difficult for neighboring cities to enjoy the dividends brought by Dig, and their development is restricted, resulting in lower Ur. At the late stage of development, the Matthew effect further emerges, the digital divide increases, and the gap between the Ur of neighboring cities and the Ur of core cities further increases^41^.

On the other hand, the development of Dig can break through the spatial barriers of traditional production, realize the optimization of production factor allocation in a larger geographical scope, reduce the waste of resources at a lower cost, improve the efficiency of factor allocation, and help the rapid and stable development of urban clusters^42^. At the same time, the development of Dig intensifies the competition and cooperation between cities, which pursue technological innovation and radiate to the surrounding cities, effectively promoting the technological development of neighboring regions. In addition, the breaking down of inter-regional information barriers also contributes to the strengthening of the overall construction of cities and jointly enhances Ur^43^. To explore what theory is more realistic, this paper presents:

### H5:

There is a spatial spillover effect in the impact of Dig on Ur.

Due to different levels of technological and economic development, the development of Dig and its impact on Ur may be different in different regions. Influenced by the initial endowment, there are differences in infrastructure, resource conditions, and policy environments in different cities, which directly lead to different levels of technological and economic development among cities^44^. Ur consists of social, economic, infrastructural, and environmental factors, and the level of science and technology and economic development play an important role in Ur.

### H6:

There is heterogeneity in the impact of Dig on Ur.

## Research design

### Sample selection and data sources

This paper takes all prefecture-level cities in China as the initial research samples and treats the samples according to the following rules: (1) excluding cities with serious missing data; (2) using interpolation to deal with samples with missing relevant variables.

The digital finance data comes from the "City Digital Inclusive Finance Index" published by the Digital Finance Center of Peking University. The rest of the data comes from the China Urban Statistical Yearbook and the statistical yearbooks published by the government websites of each city. Finally, this paper selects 252 prefecture-level cities in China from 2011 to 2020 as the research sample.

### Definition of variables

#### Urban resilience

Based on reference to the existing literature on Ur measurement, this paper constructs Ur indicators from four dimensions: economic, social, infrastructure, and ecological, and refines the categorization of the four dimensions^45^.

Economic resilience represents the economic capacity of cities in response to external shocks and is extremely important in Ur^46^. The economic dimension consists of two main aspects: endowment and structure. Economic endowment means the economic viability of a city, which directly affects the city's resistance and resilience to external changes in the economy^47^. There are four main indicators: gross domestic product (GDP) per capita, total retail sales of consumer goods, investment in fixed assets, and the number of industrial enterprises above the designated size. Economic structure refers to the economic diversity and rationality of a city's economic development, which mainly affects the city's adaptability and transformation ability after suffering from external shocks^48^. In this paper, it is expressed as the share of tertiary industry in GDP.

Social resilience is also recognized as a key element in enhancing the resilience of cities, affecting their ability to recover from external shocks^49^. Social resilience is categorized into five dimensions: stress, vitality, security, stability, and governance. Social stress includes the unemployment rate and population density. The higher the unemployment rate, the lower the resistance of the city to external shocks, while population density increases urban human capital while bringing urban spatial pressure^50^. Social vitality includes the proportion of employees in the tertiary industry and the number of university students. The stronger the overall strength of the residents, the stronger the city's ability to withstand and adapt to risks. Social security is directly related to the resilience of the city in the event of external shocks, including the number of beds, hospitals, and doctors^51^. Social stability, which includes the number of unemployed participants and the number of health insurance participants, is an important safeguard for cities to cope with shocks at the market level. In addition, at the governmental level, social governance also plays an important role in Ur, including two indicators, namely, the ratio of public budget revenues to GDP and the ratio of personnel in public administration and social organizations to the total population.

Infrastructure resilience mainly provides support services to maintain the operation of basic urban functions after a shock to the city^52^. The infrastructure dimension is constructed from two perspectives: supply and drainage. Supply includes two indicators: the electricity consumption of the whole society and the number of buses per 10,000 people. And drainage is expressed in terms of the length of the city's drainage pipes. Refined supply and drainage capacities can speed up the city's recovery and enhance its resilience efficiency^53^.

Ecological resilience represents the city's ability to cope and recover in the face of natural disasters or anthropogenic pollution risks. The ecological dimension is defined in two ways: greening and governance. Among them, the greening level is expressed by the greening coverage rate of built-up areas, and the higher the greening level, the stronger the ability of the environment itself to resist external shocks^54^. In addition to relying on the environment's own purification ability, anthropogenic environmental governance capacity also plays an important role in ecological resilience. Anthropogenic governance mainly consists of three indicators, namely the comprehensive utilization rate of industrial solid waste, the rate of harmless treatment of domestic garbage, and the centralized treatment rate of sewage treatment plants, reflecting the efforts made by human beings to enhance ecological resilience^55^.

Specific indicators under each heading are shown in Table 1.

**Table 1:** Construction of the Ur index.

First class indicator	Second class indicator	Third class indicator	Indicator properties
Economic	Economic endowment	GDP per capita	+
		Total retail sales of social consumer goods	+
		Amount of fixed asset investment	+
		Number of industrial enterprises above the scale	+
	Economic structure	The proportion of tertiary industry in GDP	+
Social	Social pressure	Unemployment rate	−
		Population density	+
	Social vitality	The proportion of tertiary industry employees	+
		University students	+
	Social security	Bed	+
		Hospital	+
		Physician	+
	Social stability	Number of unemployed participants	+
		Number of medical insurance participants	+
	Social governance	Ratio of public budget revenue to GDP	+
		Ratio of public administration and social organization personnel to total population	+
Infrastructure	Supply	Electricity consumption of the whole society	-
		Number of public buses per 10,000 people	+
	Drainage	Length of urban drainage pipeline	+
Ecology	Ecological greening	Greening coverage rate of built-up areas	+
	Ecological governance	Comprehensive utilization rate of industrial solid waste	+
		Harmless treatment rate of domestic waste	+
		Centralized treatment rate of sewage treatment plant	+

In this paper, the entropy weight TOPSIS method is used to obtain the comprehensive index of Ur. The entropy TOPSIS method synthesizes the advantages of the entropy method and TOPSIS method^56^, and the specific implementation steps are as follows:

First, standardized processing.

1 $$Y_{it} = \left\{ {_{{\frac{\max (X_{ij}) - X_{ij}}{{\max (X_{ij}) - \min (Xij)}}, \quad X_{ij}{\text{ is a negative indicator}}}}^{{\frac{X_{ij} - \min (Xij)}{\begin{subarray}{l} \max (X_{ij}) - \min (X_{ij}) \\ \end{subarray} }, \quad X_{ij}{\text{ is a positive indicator}}}} } \right.$$

In the formula, i represents the city, and j represents the measure index. x_ij_ and y_ij_ represent the index values before and after standardization, respectively.

Second, the information entropy is calculated.

2 $$E_{j} = - \frac{1}{\ln n}\sum\limits_{i = 1}^{n} {\left[ {\left( {{{Y_{ij}}/{\sum\limits_{i = 1}^{n} {Y_{ij}} }}} \right)\ln \left( {{{Y_{ij}}/{\sum\limits_{i = 1}^{n} {Y_{ij}} }}} \right)} \right]}$$

Third, construct the weighting index.

3 $$W_{j} = {{\left( {1 - E_{j}} \right)}/ {\sum\limits_{j = 1}^{m} {\left( {1 - E_{j}} \right)} }}$$

Fourth, construct the weighting matrix.

4 $$R = \left( {r_{ij}} \right)_{n \times m}$$

where, r_ij_ = W_j_*Y_ij_.

Fifth, determine the optimal and worst scheme.

5 $$Q_{j}^{ + } = \left( {\max r_{i1},\max r_{i2}, \ldots,\max r_{im}} \right)$$

6 $$Q_{j}^{ - } = \left( {\min r_{i1},\min r_{i2}, \ldots ,\min r_{im}} \right)$$

Sixth, the Euclidean distance between each scheme and the optimal and worst schemes is calculated.

7 $$d_{i}^{ + } = \sqrt {\sum\limits_{j = 1}^{m} {\left( {Q_{j}^{ + } - r_{ij}} \right)^{2} } }$$

8 $$d_{i}^{ - } = \sqrt {\sum\limits_{j = 1}^{m} {\left( {Q_{j}^{ - } - r_{ij}} \right)^{2} } }$$

Seventh, calculate the relative proximity between each measurement scheme and the ideal scheme.

9 $$C_{i} = \frac{{d_{i}^{ - } }}{{d_{i}^{ + } + d_{i}^{ - } }}$$

The higher the relative proximity of C_i_, the higher the level of resilience of the city.

#### Level of digital economy development

Regarding the measurement of the level of urban Dig development, this paper starts with two aspects: internet development and digital finance.

At the level of Internet development, four sub-items are selected to characterize the number of end-of-year cell phone subscribers, the number of Internet broadband access subscribers, the number of employees in the computer services and software industry, and the revenue from the telecommunications business^57^. Among them, two indicators—the number of end-of-year cell phone users and the number of Internet broadband access users—characterize the level of digital infrastructure construction. The good development of Dig is built on solid infrastructure, and the better the infrastructure, the more efficient the development of Dig^58^. In addition, the two indicators of the number of employees in the computer services and software industry and the revenue from telecommunication businesses represent the development of the digital industry. The larger the volume of the digital industry, the greater the potential and advantage of the future development of the digital industry, and the higher the level of Dig is^59^.

The level of digital finance, on the other hand, is indicated by the digital financial inclusion index. The index includes three dimensions, including the breadth of digital financial coverage, the depth of digital financial use, and the degree of digitization of inclusive finance, which can better reflect the level of digital finance in China^60^.

The Dig is also derived using the entropy weight TOPSIS method.

#### Mediating variables

In this paper, two mediating variables are selected: distributional effects and industrial structure upgrading.

The distributional effect is represented by the logarithm of total employee wages^31^. The development of Dig has created a new employment situation and optimized the allocation of human capital. On the one hand, the development of Dig optimizes the labor elements in traditional industries. On the other hand, the emerging industries generated by Dig also increase employment opportunities. When external shocks come, human capital represents the resistance and resilience of urban residents themselves, so this paper takes the allocation effect as one of the mediating variables. In addition, since wages are the most direct measure of the labor force, this paper uses the logarithm of total employee wages to characterize the distributional effect.

Industrial structure upgrading is characterized by the logarithm of industrial value added^61^. Traditional industries are developed through intelligentization and informatization, which ultimately achieve the optimization of resource allocation as well as the improvement of production efficiency. At the same time, the rapid development of cloud computing, big data, and other emerging technologies has gradually formed a new high-tech industry centered on technology and information. The combination of the two ultimately results in the upgrading of industrial structures. And a good industrial structure in turn promotes economic development, social stability, and the strengthening of infrastructure construction in the city and reduces pollution in the environment. Therefore, this paper adopts industrial structure upgrading as the mediating variable. The value added of industry reflects, to some extent, the transformation of the production process from low-value products to high-value products. Therefore, this paper uses the proportion of industrial added value to regional GDP to characterize the upgrading of industrial structures.

#### Control variables

Four control variables are selected in this paper: foreign capital use, financial development level, share of science and education inputs, and urbanization rate.

Foreign capital use is an important indicator of Ur, characterized by the logarithm of the amount of foreign capital actually used in the year^62,63^. On the one hand, the increase in the use of foreign capital is conducive to promoting the development of domestic enterprises towards diversification, increasing economic vitality, and enhancing the economic resilience of the city. On the other hand, the increase in the use of foreign capital may bring high-consumption and high-pollution industries, reducing the city's ecological resilience.

The level of financial development is characterized by the year-end deposit and loan balances of financial institutions over GDP^64^. The year-end deposit and loan balances represent the idle social funds of the city, and the larger the ratio of year-end deposit and loan balances to GDP, the more idle social funds there are. By expanding the fiscal scale, these funds can be aggregated and used to enhance the city's infrastructure, improving the city's economic resilience and infrastructure resilience.

The ratio of investment in science and education is characterized by the proportion of science and education expenditure to local public budget expenditure, which represents the innovation activity of a city^65^. Enhancing investment in science and education can optimize industrial structure, promote economic growth, and improve the economic resilience of cities. At the same time, investment in science and education can also improve the quality of the urban labor force and enhance urban social resilience.

Urbanization rate Urbanization is measured using the proportion of non-agricultural population to the total population of the region at the end of the year^66^. On the one hand, the higher the urbanization rate, the greater the consumption of urban resources, which is prone to resource crises. On the other hand, an increase in the urbanization rate reduces the population density in the countryside, which helps the recovery of forests, grasslands, and other vegetation.

### Model setting

#### Benchmark model

To test the impact of Dig on Ur, we constructed the following model (10):

10 $$Ur_{it} = \alpha + \beta Dig_{it} + \gamma Controls_{it} + \mu_{i} + \delta_{t} + \varepsilon_{it}$$

The dependent variable Ur_it_ represents the level of resilience of the i city in year t. Dig_it_ is the core explanatory variable, representing the level of Dig of the i city in year t. Controls_it_ is a set of control variables. This paper uses robust standard errors to estimate the regression model.

#### Channel and mechanism modeling

This paper uses a mediated effects model to test the role of distributional effects and industrial structure upgrading in the mechanisms by which Dig affects Ur. The full-mediated effects model is:

11 $$Ur_{it} = a_{0} + {\text{ a}}_{1} Dig_{it} + \beta_{1} Controls_{it} + \mu_{i} + \delta_{t} + \varepsilon_{it}$$

12 $$T_{it} = b_{0} + \, b_{1} Dig_{it} + \beta_{2} Controls_{it} + \mu_{i} + \delta_{t} + \varepsilon_{it}$$

13 $$Ur_{it} = \, c_{0} + \phi T_{it} + \, c_{1} Dig_{it} + \beta_{3} Controls_{it} + \mu_{i} + \delta_{t} + \varepsilon_{it}$$

where T denotes the mediating variable.

#### MMQR model

In order to deeply explore the impact of digital economic development on Ur, this paper uses the MMQR model for further analysis^67,68^. The following model is used to quantify the conditional quantum location scale Qy(τ|R) variable:

14 $$Y_{it} = \gamma_{i} + \alpha R_{it} + \, (\sigma_{i} + \varphi X_{it} )\mu_{it}$$

The probabilities are determined by p(σ_i_ + φX′_it_ > 0) = 1, while the predictor coefficients are γ, α, σ, and φ. In addition, the k-vector of standard elements of R is denoted by X, which is the characteristic variation with component l, as shown in model (15):

15 $$X_{l} = X_{l} (R), \quad l = 1,2, \ldots ,k$$

where R_it_ is uniformly and independently distributed for all fixed i and t. The general form of model (14) can be written as model (16):

16 $$Q_{y} (\tau |R_{it} ) \, = \, (\gamma_{i} + \sigma_{i} q(\tau )) \, + \alpha R_{it} + \varphi X{^{\prime}}_{it} q(\tau )$$

R_it_ in model (16) is a vector of explanatory variables, including Dig and a set of control variables. And q(τ) represents the τ sample of the quantile, and four samples were evaluated in this study, namely the 25th, 50th, 75th, and 90th. Therefore, the quantile equation in this study is shown in model (17) as follows:

17 $$min_{q} \sum\limits_{i}^{{}} {\sum\limits_{t}^{{}} {\delta_{\tau } } } (R_{it} - \, (\sigma_{i} + \varphi X{^{\prime}}_{it} ) \cdot q)$$

#### Threshold model

The nonlinear effect between Dig and Ur is further examined by a threshold regression model. Among them, the bootstrap method was used to repeat the sampling 300 times to estimate the threshold. The specific model is as follows:

18 $$Ur_{it} = {\text{ f}}_{0} + f_{1} Dig_{it} I\left( {M_{it} \le Z_{1} } \right) + f_{2} Dig_{it} I\left( {Z_{1} < M_{it} \le Z_{2} } \right) + \cdots + f_{n + 1} Dig_{it} I\left( {M_{it} > Z_{n} } \right) + \mu_{P} + \delta_{I} + \lambda_{Y} + \varepsilon_{it}$$

#### Spatial spillover effects

A spatial model (19) is established to further explore the spatial effects of the impact of Dig on Ur, where $\rho$ is the spatial auto-regressive coefficient and W is the spatial weight matrix.

19 $$Ur_{it} = \alpha_{1} + \rho WUr_{it} + \eta_{1} WDig_{it} + \eta_{2} Dig_{it} + \gamma_{1} WControl_{it} + \gamma_{2} Control_{it} + \mu_{i} + \delta_{t} + \varepsilon_{it}$$

In this paper, the geographic inverse distance spatial weight matrix W_1_ and the geographic inverse distance squared spatial weight matrix W_2_ are chosen, and they are calculated as follows:

20 $$W_{1} = \left\{ {\begin{array}{ll} {1/d_{{ij}} } & \quad {i \ne j} \\ 0 & \quad {i = j} \\ \end{array} } \right.$$

21 $$W_{1} = \left\{ {\begin{array}{ll} {1/d^{2}{_{{ij}}} } & \quad {i \ne j} \\ 0 & \quad {i = j} \\ \end{array} } \right.$$

## Results

### Baseline regression and robustness analysis

#### Descriptive statistics

Table 2 reports the descriptive statistics of the main variables. In terms of Ur, the standard deviation of Ur level in China (0.062) is small, indicating that the Ur level of most cities in China is close to the average (0.072), and there is not much difference in Ur level nationwide. In terms of Dig development level, the Dig level of the head city (0.797) is much higher than the national average (0.052), which shows that the radiation effect of Dig has not yet been fully released on a national scale.

**Table 2:** Descriptive statistics of the main variables.

Variable	Symbol	Variable name	Sample number	Mean value	Standard deviation	Minimum value	Maximum value
Dependent variable	Ur	Urban resilience	2520	0.072	0.062	0.017	0.658
Core explanatory variable	Dig	Urban digital economy development level	2520	0.052	0.062	0.002	0.797
Mediating variable Control variable	De	Distributive effect	2500	5.322	1.008	2.105	9.489
	Is	Upgrading of industrial structure	2517	6.602	0.966	3.503	9.266
Variable	Lfd	Use of foreign capital	2520	2.458	1.220	0.588	21.302
	Afc	Financial development level	2520	10.058	2.368	− 9.210	14.941
	Ei	Proportion of investment in science and education	2520	0.195	0.043	0.006	0.372
	Uz	Urbanization rate	2520	0.423	0.253	0.107	1.926

#### Baseline analysis and robustness tests

Column (1) of Table 3 shows the results of the baseline regression on the impact of Dig on Ur. The results show that the impact of Dig on Ur is positive and significant, and Hypothesis 1 is tested.

**Table 3:** Benchmark analysis and robustness test.

	(1) Ur	(2) Ur	(3) Ur	(4) Ur	(5) Ur2	(6) Ur	(7) Ur	(8) Ur
Dig	0.757*** (0.112)				0.690*** (0.094)			1.318*** (0.249)
L.Dig		0.763*** (0.125)	0.759*** (0.124)					
L2.Dig				0.753*** (0.157)				
Dig2						0.777*** (0.078)		
Dig3							0.026*** (0.003)	
Controls	Yes	Yes	Yes	Yes	Yes	Yes	Yes	Yes
Year FE	Yes	Yes	Yes	Yes	Yes	Yes	Yes	Yes
City FE	Yes	Yes	Yes	Yes	Yes	Yes	Yes	Yes
First stage F-test								22.18***
Kleibergen-Paap rk LM statistics								17.833***
N	2520	2268	2268	2016	2520	2520	2520	2268
R^2^	0.932	0.937	0.937	0.941	0.959	0.938	0.928	0.923

Note: *** p<0.01.

The baseline regression results may be challenged by potential endogeneity issues. On the one hand, this paper adopts the lagged variable approach to mitigate the endogeneity problem. On the other hand, robustness tests are conducted by replacing the dependent and core explanatory variables. Finally, the paper also utilizes instrumental variables to conduct further tests.

First, one-period lags are applied to all explanatory variables of the baseline model, and the results are shown in column (2) of Table 3. Second, one- and two-period lagged regressions are performed on the core explanatory variables, respectively, and the results are shown in columns (3) and (4) of Table 3. Third, the entropy weight method is used to measure the resilience of Chinese cities. The results are shown in column (5) of Table 3. Fourth, the entropy weight method and principal component analysis are used to measure Dig composite index with replacement analysis, and the results are shown in columns (6) and (7) of Table 3.

Meanwhile, the lagged period of Dig is selected as an instrumental variable in this paper. The two-stage least squares method (2SLS) was used for the estimation. The results show that the Kleibergen-Paap rk LM statistics of the second stage significantly prove that the instrumental variables are correlated with the endogenous variables. Moreover, the F statistic of the first-stage regression is greater than 10, and the instrumental variables are valid. Column (8) of Table 3 shows the final results of the instrumental variable tests.

In summary, the reliability and robustness of Hypothesis 1 are verified, and Dig effectively contributes to Ur.

It is not surprising that hypothesis 1 has been proven. In fact, the development of digital financial inclusion has had a significant effect on China's economic vitality. At the same time, "smart cities" have improved the efficiency of social operations and optimized the construction of infrastructure. And with the application of more digital technologies in low-carbon areas, the urban ecological environment has also been significantly improved. All in all, Dig is influencing Ur in all aspects.

### Channel and mechanism analysis

In this paper, we conduct channel and mechanism tests for distributional effects and industrial structure upgrading, and the test results are shown in Table 4.

**Table 4:** Mechanistic test of Dig on Ur.

	(1) Ur	(2) De	(3) Ur	(4) Ur	(5) Is	(6) Ur
Dig	0.757*** (0.112)	0.876*** (0.225)	0.739*** (0.111)	0.757*** (0.112)	0.612** (0.239)	0.755*** (0.112)
De			0.008*** (0.002)			
Is						0.003* (0.002)
Controls	Yes	Yes	Yes	Yes	Yes	Yes
Year FE	Yes	Yes	Yes	Yes	Yes	Yes
City FE	Yes	Yes	Yes	Yes	Yes	Yes
N	2520	2500	2500	2520	2517	2517
R2	0.932	0.961	0.933	0.932	0.969	0.932

Note: *** p<0.01, ** p<0.05, * p<0.1.

Column (2) of Table 4 shows that Dig can positively promote De, and column (3) shows that the effects of Dig and De on Ur are all positive and significant. Columns (2) and (3) clarify the mediating role of De with a mediating effect of 0.018, which accounts for 2.38% of the total effect, and Hypothesis 2 is tested. Similarly, columns (5) and (6) of Table 4 show that Dig positively and significantly improves Ur by promoting industrial structure upgrading, and Hypothesis 3 is tested.

Hypotheses 2 and 3 are verified, clarifying the path of Dig affecting Ur. On the one hand, Dig optimizes urban distribution, stimulates urban innovation, improves the unemployment rate, enhances social stability, and thus raises Ur. On the other hand, Dig reduces the vulnerability of the urban system by promoting the development of advanced industries, improving the structure of backward industries, and guiding the city in the direction of knowledge and technology-intensive development, thus raising Ur.

### MMQR analysis

The normality test and cointegration test tables for the data are shown in the Appendix (Supplementary Tables 1–4), and the results indicate that the data are non-normal and that there is a cointegration relationship between all the explanatory variables. In this paper, the MMQR model that can handle non-normally distributed data is used for analysis, and the results are shown in Table 5.

**Table 5:** MMQR test.

Ur	Quantiles					
	Location	Scale	0.25	0.50	0.75	0.90
Dig	0.645*** (8.59)	0.196*** (3.22)	0.458*** (7.45)	0.607*** (8.96)	0.774*** (7.54)	0.963*** (6.26)

Note: *** p<0.01.

The results show that the effect of Dig on Ur is positive and significant in all quartiles. In addition, Q0.25, Q0.50, Q0.75, and Q0.90 gradually increase the contribution to Ur from low to high scores. The higher the level of Ur, the greater the contribution of Dig to the improvement of Ur.

After testing the data for slope heterogeneity and cross-sectional dependence, the results are further analyzed in this paper using the Dumitrescu and Hurlin causality tests. The results of the tests are presented in the appendix (Supplementary Table 5). The results of the MMQR regressions allow Hypothesis 4 to be argued.

### Nonlinear threshold effect

Based on Hypothesis 4, this paper attempts to further investigate the critical threshold value of the nonlinear effect of Dig on Ur. A threshold regression model is used to estimate the threshold value by applying the bootstrap method with 300 repeated samples. The test results of the threshold effect and threshold value are shown in the appendix (Supplementary Table 6), and the regression results of the threshold model are shown in Table 6.

**Table 6:** Regression results of the threshold model.

	Ur(1)	Ur(2)	Ur(3)
Threshold	Dig ≤ 0.026	0.026 < Dig ≤ 0.082	Dig > 0.082
Dig	0.960*** (0.071)	0.576*** (0.031)	0.765*** (0.019)

Note: *** p<0.01.

The threshold analysis leads to the following conclusions: First, Dig has a significant dynamic nonlinear spillover effect on Ur enhancement, and Hypothesis 4 is further verified. Second, Dig has a double threshold effect on Ur enhancement. When the level of Dig crosses the first threshold, the spillover effect is weakened, while when the second threshold is crossed, the spillover effect is enhanced. The impact of Dig on Ur enhancement shows a U-shaped change trend.

The argument for Hypothesis 4 brings further thoughts. From the perspective of Ur, since the higher the Ur, the more significant the effect of Dig development on Ur is, then cities with high Ur should vigorously promote Dig development. The Matthew effect of "the strong continue to be strong and the strong get stronger" is very clear here.

From the perspective of Dig, as the level of Dig continues to increase, the promotion effect of Dig on Ur shows a U-shaped change. The possible explanation is that in the early stages of Dig development, such as the arrival of the "Internet Plus Era" and the "Big Data Era", the effects of Dig on all aspects of the city are immediately visible. However, on the one hand, based on the principle of diminishing marginal utility, on the other hand, the development of digital technology has also brought many negative effects. For example, on the economic side, "digital monopoly" and "digital hegemony" are becoming more and more prominent; on the social side, digital security has also become a major hidden danger that cannot be ignored; on the infrastructure and ecological side, behind the powerful computing power of digital technology, its consumption of equipment and electricity brings environmental pollution problems that cannot be ignored. Development is accompanied by a variety of problems. Fortunately, with the growing maturity of digital technology, the above problems have been improved to varying degrees. The government has more and more experience and maturity in dealing with the problems brought about by Dig. At the same time, with the continuous breakthroughs in the bottleneck of digital technology, Dig's "Metcalfe" effect will be more and more significant, and its impact on the Ur will be further enhanced.

### Spatial spillover effects

The results of the spatial correlation test show that the Moran I indexes of Dig and Ur are both positive, and the corresponding Moran I indexes are significant at the 5% level in most of the years, as detailed in the Appendix (Supplementary Table 7). This indicates that there is a strong positive spatial autocorrelation for both Dig and Ur and justifies the choice of the spatial regression model in this paper. The SDM spatial model with double fixed effects was selected through the LM test, Hausman test, Wald test, and LR test and determined under two weight matrices. Table 7 reports the results of estimating spatial effects under different spatial weight matrices.

**Table 7:** Spatial effects under different spatial weight matrices.

	Ur	Ur
	W1 (SDM)	W2 (SDM)
Dig	0.765*** (0.024)	0.748*** (0.024)
w*Dig	1.065*** (0.394)	0.005 (0.111)
rho	0.614*** (0.100)	0.479*** (0.047)
Direct effect	0.782*** (0.026)	0.761*** (0.024)
Indirect effect	4.605* (2.489)	0.707*** (0.199)
Total effect	5.387** (2.500)	1.468*** (0.207)
Control variables	Yes	Yes
City FE	Yes	Yes
Year FE	Yes	Yes
N	2520	2520
R^2^	0.733	0.759

Note: *** p<0.01, ** p<0.05, * p<0.1.

The coefficients on Dig and w*Dig in Table 7 show the point estimate effects of spatial spillovers, and in order to address the problem of estimation bias that can result from point estimates^69^, it is necessary to decompose the total effect into direct and indirect effects. Direct spillovers show the effect of an increase in the level of Dig on the Ur of the region, and indirect spillovers show the effect of Dig growth on the Ur of neighboring regions.

As shown in Table 7, in the two different spatial weight matrices, the SDM model shows significant positive direct and total effects, which indicates that Dig can effectively improve Ur and promote urban resilience, and Hypothesis 1 is further verified. The indirect effect is also significant positive, which shows that Dig can have an impact on Ur in nearby areas through spatial spillover effects. This proves Hypothesis 5.

The argument of Hypothesis 5 identifies the spatial spillover effects of Dig and Ur. On the one hand, there are significant spatial effects of Dig and Ur themselves. A city's development must have an impact on its neighboring areas, and the level of Ur itself cannot be discussed in isolation from the geographical location factor because economic development, social operation, ecological environment, and other factors have synergistic effects in themselves.

On the other hand, there is a significant spatial effect of Dig on Ur. Dig development in a region not only affects the local Ur level but also contributes to the Ur level of neighboring regions.

### Heterogeneity analysis

First, based on the "City Business Attractiveness Ranking" published by China's "First Finance", first-tier, new first-tier, second-tier, and third-tier cities are categorized as high-class cities, while other cities are categorized as low-class cities^39^. Secondly, the cities are categorized into a sample of focused cities and a sample of non-focused cities. According to the Chinese government's specific classification of urban agglomerations in each region of China, this paper categorizes the Beijing-Tianjin-Hebei delta, the Yangtze River delta, the Pearl River delta, the middle reaches of the Yangtze River, the Central Plains, and the Chengdu-Chongqing urban agglomerations as the sample of key urban agglomerations, and the others as non-focused. Finally, the sample is categorized by region into East, West, Central, and Northeast according to the National Bureau of Statistics of China (NBS) economic zones. Details of the heterogeneous city list categorization are shown in the appendix (Supplementary Tables 8–10).

As a matter of fact, this paper discusses heterogeneity at the three levels of city classes, city clusters, and regions based on the following considerations:

First, Caijing First classifies Chinese cities according to five aspects: business resources, transportation hubs, resident activity, lifestyles, and future plasticity, and the higher the class, the higher the overall endowment. In this paper, cities with higher comprehensive endowments have higher Ur accordingly, and the promotion effect of Dig on Ur may not be more significant. Based on the perspective of city class, this paper tries to explore the promotion effect of Dig on Ur in cities with different levels of comprehensive endowment.

Second, an urban agglomeration is an organic aggregate of multiple cities constituted by a central city as the core and radiating around it. City clusters are closely linked economically, united in terms of social operation, practicing co-construction through urban planning and infrastructure, and ecologically influencing each other. Based on the perspective of urban agglomeration, this paper attempts to explore the comprehensive impact of Dig on the resilience of "urban agglomerations" under the pattern of synergistic development among multiple cities.

Third, while the previous two points mainly focused on cities and urban agglomerations, this paper tries to categorize Chinese cities into four major segments at a more macro-regional level and explore the differences between the segments.

#### Heterogeneity analysis of direct effects

Results by class are shown in columns (1)–(2) of Table 8. The results of the heterogeneity of direct effects by urban agglomeration are shown in columns (3)–(4) of Table 8. The results by region are shown in columns (5)–(8) of Table 8.

**Table 8:** Heterogeneity test.

	(1) Ur	(2) Ur	(3) Ur	(4) Ur	(5) Ur	(6) Ur	(7) Ur	(8) Ur
Dig	0.642*** (0.103)	0.140** (0.058)	0.798*** (0.125)	0.140 (0.118)	0.757*** (0.156)	0.632*** (0.132)	0.645*** (0.147)	0.297 (0.604)
Controls	Yes	Yes	Yes	Yes	Yes	Yes	Yes	Yes
Year FE	Yes	Yes	Yes	Yes	Yes	Yes	Yes	Yes
City FE	Yes	Yes	Yes	Yes	Yes	Yes	Yes	Yes
N	1170	1350	1140	1380	850	640	740	290
R^2^	0.933	0.667	0.947	0.870	0.945	0.897	0.916	0.891

Note: *** p<0.01.

The results show that the impact of Dig on Ur is greater in high-class cities, key city clusters, and eastern regions. On the one hand, Dig can better promote Ur in cities with higher comprehensive endowments, indicating that the higher comprehensive endowment can promote the release of the role of Dig. On the other hand, the effect of Dig on Ur is greater in the synergistic "city agglomerations", which shows that the role of Dig releases a greater effect in the synergistic effect of cities. Meanwhile, looking at the regional segments, Dig in the eastern region promotes Ur more, showing that the digital economy in the coastal region has greater influence.

#### Heterogeneity analysis of nonlinear spillover effects

The heterogeneity analysis of nonlinear spillovers is detailed in the Appendix (Supplementary Tables 11 and 12).

The results show that, first, cities in high-class cities, key city clusters, eastern regions, and western regions pass the threshold test, further proving that the promotion effect of Dig on Ur is more significant in cities with high comprehensive endowments, city clusters, and coastal regions. Second, from the overall trend, the nonlinear effect of Dig shows a tendency toward decreasing utility.

Combining direct effect analysis and nonlinear effect analysis, it can be seen that the impact of Dig on Ur is heterogeneous, and hypothesis 6 is verified.

## Conclusions and recommendations

Taking China as an example, this paper systematically explores the impact mechanism of Dig on China's Ur in three dimensions: linear effect, nonlinear effect, and spatial effect, based on data from 252 cities from 2011 to 2020 and eight sets of samples from city classes, city clusters, and different regions. This study can provide some ideas and inspiration for cities in various countries to enhance Ur and promote sustainable urban development through the development of Dig.

First, the development of Dig should be accelerated. This paper concludes that the development of Dig can contribute significantly to the enhancement of Ur. Consistent with Agboola's study of urban resilience in Nigeria in the digital era, by collecting and analyzing data in real time, timely adjustments can be made to urban development strategies for maximum efficiency and sustainability. This not only contributes to resource conservation but also enhances Nigeria's overall resilience and adaptability in the face of external risks^70^. Therefore, in order to pursue sustainable urban development, Dig should be seized as a key factor. Specifically, the construction of digital infrastructure should be strengthened first. Digital infrastructure is the cornerstone of Dig, and government departments should increase corresponding construction investment to ensure the stable development of Dig. Secondly, industries should seize the opportunity to promote digital industrialization and industrial digitalization to ensure the long-term stable development of Dig.

Secondly, the role of the urban distribution effect and industrial structure upgrading in Dig to enhance Ur should be emphasized. From the perspective of the channel mechanism, the development of Dig can improve Ur by optimizing the urban distribution effect and promoting the upgrading of urban industrial structures. On the one hand, cities should actively use digital technology to optimize the allocation of production factors. In particular, they should strengthen the utilization efficiency of human capital, improve the fairness and reasonableness of distribution, form a virtuous cycle, and gradually enhance Ur. On the other hand, cities should actively encourage the development of emerging digital industries and improve industrial diversity. At the same time, cities should promote the combination of digital technology and traditional industries to promote the development of cities in the direction of technology-intensive development and enhance the overall resilience of cities.

Third, the focus is on grasping the non-linear impact relationship between Dig and Ur. On the one hand, cities with higher quartiles of Ur should more vigorously develop Dig. On the other hand, the impact of Dig on Ur has an obvious threshold effect, showing a "U" trend. Chatti's findings are similar to this conclusion, suggesting that the environmental impact of digital technology is greater in developed countries than in developing countries^71^. The non-linear relationship requires cities to pay attention to their own specific development stages and formulate development paths that are differentiated by stage. Specifically, while Dig can positively enhance Ur, prioritizing a comprehensive and long-term development plan is the right way to maximize benefits and achieve sustainable growth.

Fourthly, attention should be paid to the spatial spillover effect of the impact of Dig on Ur. The emergence of Dig is both an opportunity and a challenge, and the digital divide is an issue that should be a concern in spatial effects, which should be addressed to ensure that every city does not fall behind and that the goal of sustainable development can be realized. On the one hand, there is a strong spatial autocorrelation between the levels of Dig and Ur. On the other hand, Dig can effectively enhance the resilience of a city and affect neighboring cities through spatial spillover effects. This conclusion is supported by Hao, who argues that there is a significant spatial synergy effect of Dig on urban sustainability, which is manifested in the spatial spillover effect of "1 + 1 > 2"^72^. Based on this, the synergistic development strategy between cities should be rationally planned at the macro level, and the development of Dig and the pursuit of Ur should fully take into account the development status of neighboring cities and be integrated into the overall planning system. At the same time, it is possible to establish city clusters for the synergistic development of Dig and Ur, to strengthen information interoperability and resource sharing, and to promote cooperation and competition between neighboring cities.

Fifth, grasp the heterogeneous impact of Dig on Ur. In the heterogeneity test, the impact of Dig on Ur is more obvious in high-class cities, key city clusters, and eastern regions. At the same time, the nonlinear spillover effect of Dig is released more fully in high-class cities, key city clusters, and cities in eastern and western regions. Jiang's research supports this conclusion, and in cities with higher levels of comprehensive development, such as those in the East, cities should capitalize on their own economic resource endowments to move forward with all-round digital transformation more quickly and with higher quality^73^. On this basis, the paper proposes that cities should fully and accurately recognize their own spatial characteristics and formulate differentiated digital economies and urban development strategies. Specifically, areas where Dig has a better impact on Ur, such as high-class and key cities or eastern regions, should focus on technological breakthroughs in Dig, strengthen investment in high-tech technology research and development, and pursue a higher impact effect. On the contrary, existing cities that are less developed should strengthen infrastructure construction and promote Dig and resilience development on a regular basis as soon as possible.

Although this paper has tried its best to pursue a complete analysis, the article inevitably has some limitations. Firstly, both "Dig" and "Ur" are in the stage of rapid development, and the connotation and extension of the two may change at any time. The research scope adopted in this paper may have timeliness, and the indicators of "Dig" and "Ur" involved in future research may be more comprehensive. Secondly, based on the existing theoretical foundation, this paper has only selected two mechanism variables, namely, urban distribution effect and industrial structure upgrading, while there are many potential variables in practice, such as digital governance, etc., which may be explored more in future research. Finally, although the study includes comparative references to similar studies abroad, the generalizability of the conclusions is limited by the fact that the data related to Chinese cities are only analyzed, and future studies can analyze city samples from multiple countries to discuss Dig and Ur in a larger geographical scope.

### Supplementary Information

Supplementary Information.

## Data Availability

The data used in this study are available upon request from the corresponding author.

## References

[1] Cañavera-Herrera JS, Tang J, Nochta T, Schooling JM (2022). On the relation between ‘resilience’ and ‘smartness’: A critical review. Int. J. Disaster Risk Reduct..

[2] de Jong M, Joss S, Schraven D, Zhan C, Weijnen M (2015). Sustainable–smart–resilient–low carbon–eco–knowledge cities; making sense of a multitude of concepts promoting sustainable urbanization. J. Clean Prod..

[3] Hatuka T, Rosen-Zvi I, Birnhack M, Toch E, Zur H (2018). The political premises of contemporary urban concepts: The global city, the sustainable city, the resilient city, the creative city, and the smart city. Plan. Theory Pract..

[4] Shamsuddin S (2020). Resilience resistance: The challenges and implications of urban resilience implementation. Cities..

[5] Ribeiro PJG, Pena Jardim Gonçalves LA (2019). Urban resilience: A conceptual framework. Sustain. Cities Soc..

[6] Bueno S, Bañuls VA, Gallego MD (2021). Is urban resilience a phenomenon on the rise? A systematic literature review for the years 2019 and 2020 using textometry. Int. J. Disaster Risk Reduct..

[7] Ye C, Hu M, Lu L, Dong Q, Gu M (2022). Spatio-temporal evolution and factor explanatory power analysis of urban resilience in the Yangtze river economic belt. Geogr. Sustain..

[8] Jiang N, Jiang W (2024). How does regional integration policy affect urban resilience? Evidence from urban agglomeration in China. Environ. Impact Assess. Rev..

[9] Datola G (2023). Implementing urban resilience in urban planning: A comprehensive framework for urban resilience evaluation. Sustain. Cities Soc..

[10] Anelli D, Tajani F, Ranieri R (2022). urban resilience against natural disasters: Mapping the risk with an innovative indicators-based assessment approach. J. Clean Prod..

[11] Milios L (2018). Advancing to a circular economy: Three essential ingredients for a comprehensive policy mix. Sustain. Sci..

[12] Arbolino R, De Simone L, Carlucci F, Yigitcanlar T, Ioppolo G (2018). Towards a sustainable industrial ecology: Implementation of a novel approach in the performance evaluation of Italian regions. J. Clean Prod..

[13] Ingrao C, Messineo A, Beltramo R, Yigitcanlar T, Ioppolo G (2018). How can life cycle thinking support sustainability of buildings? Investigating life cycle assessment applications for energy efficiency and environmental performance. J. Clean Prod..

[14] DAmico G, Arbolino R, Shi L, Yigitcanlar T, Ioppolo G (2022). Digitalisation driven urban metabolism circularity: A review and analysis of circular city initiatives. Land Use Pol..

[15] De Pascale A, Arbolino R, Szopik-Depczyńska K, Limosani M, Ioppolo G (2021). A systematic review for measuring circular economy: The 61 indicators. J. Clean Prod..

[16] Du Y, Wang Q, Zhou J (2023). How does digital inclusive finance affect economic resilience: Evidence from 285 cities in China. Int. Rev. Financ. Anal..

[17] Yu Z, Li Y, Dai L (2023). Digital finance and regional economic resilience: Theoretical framework and empirical test. Financ. Res. Lett..

[18] Lei Y, Liang Z, Ruan P (2023). Evaluation on the impact of digital transformation on the economic resilience of the energy industry in the context of artificial intelligence. Energy Rep..

[19] Pee LG, Pan SL (2022). Climate-intelligent cities and resilient urbanisation: Challenges and opportunities for information research. Int. J. Inf. Manag..

[20] Campioni L (2023). Enabling civil-military collaboration for disaster relief operations in smart city environments. Future Gen. Comput. Syst..

[21] Garcia-Perez A, Cegarra-Navarro JG, Sallos MP, Martinez-Caro E, Chinnaswamy A (2023). Resilience in healthcare systems: Cyber security and digital transformation. Technovation..

[22] Lin B, Huang C (2023). How will promoting the digital economy affect electricity intensity?. Energy Policy..

[23] Xu S, Wang X, Zhu R, Wang D (2023). Spatio-temporal effects of regional resilience construction on carbon emissions: Evidence from 30 Chinese provinces. Sci. Total Environ..

[24] Hudec O, Reggiani A, Šiserová M (2018). Resilience capacity and vulnerability: A joint analysis with reference to Slovak urban districts. Cities..

[25] Pan W, Xie T, Wang Z, Ma L (2022). Digital economy: An innovation driver for total factor productivity. J. Bus. Res..

[26] Guo B (2023). Impact of the digital economy on high-quality urban economic development: Evidence from Chinese cities. Econ. Model..

[27] Asongu S, Amari M, Jarboui A, Mouakhar K (2021). ICT dynamics for gender inclusive intermediary education: Minimum poverty and inequality thresholds in developing countries. Telecommun. Policy..

[28] Dutta UP, Gupta H, Sengupta PP (2019). ICT and health outcome nexus in 30 selected Asian countries: Fresh evidence from panel data analysis. Technol. Soc..

[29] Zhu Q, Zhu C, Peng C, Bai J (2022). Can information and communication technologies boost rural households’ income and narrow the rural income disparity in China?. China Econ. Q. Int..

[30] Avom D, Nkengfack H, Fotio HK, Totouom A (2020). ICT and environmental quality in sub-Saharan Africa: Effects and transmission channels. Technol. Forecast. Soc. Chang..

[31] Zhou Q, Zhu M, Qiao Y, Zhang X, Chen J (2021). Achieving resilience through smart cities? Evidence from China. Habitat Int..

[32] Laudien SM, Pesch R (2019). Understanding the influence of digitalization on service firm business model design: A qualitative-empirical analysis. Rev. Manag. Sci..

[33] Chang H, Ding Q, Zhao W, Hou N, Liu W (2023). The digital economy, industrial structure upgrading, and carbon emission intensity—Empirical evidence from China's provinces. Energy Strateg. Rev..

[34] Mo C, He C, Yang L (2020). Structural characteristics of industrial clusters and regional innovation. Econ. Lett..

[35] Tang D, Li J, Zhao Z, Boamah V, Lansana DD (2023). The influence of industrial structure transformation on urban resilience based on 110 prefecture-level cities in the Yangtze river. Sustain. Cities Soc..

[36] Bai L (2023). Effects of digital economy on carbon emission intensity in Chinese cities: A life-cycle theory and the application of non-linear spatial panel smooth transition threshold model. Energy Policy..

[37] Cheng Y, Zhang Y, Wang J, Jiang J (2023). The impact of the urban digital economy on China's carbon intensity: Spatial spillover and mediating effect. Resour. Conserv. Recycl..

[38] Contador JC, Satyro WC, Contador JL, Spinola MDM (2021). Taxonomy of organizational alignment: Implications for data-driven sustainable performance of firms and supply chains. J. Enterp. Inf. Manag..

[39] Ma D, Zhu Q (2022). Innovation in emerging economies: Research on the digital economy driving high-quality green development. J. Bus. Res..

[40] Liu J, Yu Q, Chen Y, Liu J (2022). The impact of digital technology development on carbon emissions: A spatial effect analysis for China. Resour. Conserv. Recycl..

[41] Gómez-Bengoechea G, Jung J (2024). The Matthew effect: Evidence on firms’ digitalization distributional effects. Technol. Soc..

[42] Ren S, Hao Y, Xu L, Wu H, Ba N (2021). Digitalization and energy: How does internet development affect China's energy consumption?. Energy Econ..

[43] Yu H, Wang J, Xu J (2023). Assessing the role of digital economy agglomeration in energy conservation and emission reduction: Evidence from China. Energy..

[44] Wang J, Wang B, Dong K, Dong X (2022). How does the digital economy improve high-quality energy development? The case of China. Technol. Forecast. Soc. Chang..

[45] Liu Y, Han L, Pei Z, Jiang Y (2023). Evolution of the coupling coordination between the marine economy and urban resilience of major coastal cities in China. Mar. Pol..

[46] Rahman MA (2020). Data-driven dynamic clustering framework for mitigating the adverse economic impact of covid-19 lockdown practices. Sustain. Cities Soc..

[47] Zhu Z, Zheng Y, Xiang P (2023). Deciphering the spatial and temporal evolution of urban anthropogenic resilience within the Yangtze river delta urban agglomeration. Sustain. Cities Soc..

[48] Cutter SL, Ash KD, Emrich CT (2014). The geographies of community disaster resilience. Glob. Environ. Change..

[49] Saja AMA, Teo M, Goonetilleke A, Ziyath AM (2018). An inclusive and adaptive framework for measuring social resilience to disasters. Int. J. Disaster Risk Reduct..

[50] Shi C, Guo N, Gao X, Wu F (2022). How carbon emission reduction is going to affect urban resilience. J. Clean Prod..

[51] Liu L (2022). The Spatio-temporal dynamics of urban resilience in China's capital cities. J. Clean Prod..

[52] Kontokosta CE, Malik A (2018). The resilience to emergencies and disasters index: Applying big data to benchmark and validate neighborhood resilience capacity. Sustain. Cities Soc..

[53] Chen, Y., Zhu, M., Zhou, Q. & Qiao, Y. Research On Spatiotemporal Differentiation and Influence Mechanism of Urban Resilience in China Based On Mgwr Model. *Int. J. Environ. Res. Public Health*. **18**, 1056 (2021). 10.3390/ijerph18031056 PMC7908263 33504043

[54] Zhao R, Fang C, Liu J, Zhang L (2022). The evaluation and obstacle analysis of urban resilience from the multidimensional perspective in Chinese cities. Sustain. Cities Soc..

[55] Datola G, Bottero M, De Angelis E, Romagnoli F (2022). Operationalising resilience: A methodological framework for assessing urban resilience through system dynamics model. Ecol. Model..

[56] Sun Y, Wang Y, Zhou X, Chen W (2023). Are shrinking populations stifling urban resilience? Evidence from 111 resource-based cities in China. Cities..

[57] Huang L, Zhang H, Si H, Wang H (2023). Can the digital economy promote urban green economic efficiency? Evidence from 273 cities in China. Ecol. Indic..

[58] Wu J, Lin K, Sun J (2023). Improving urban energy efficiency: What role does the digital economy play?. J. Clean Prod..

[59] Wang L, Shao J (2023). Digital economy, entrepreneurship and energy efficiency. Energy..

[60] Guo F (2020). Measuring China's digital financial inclusion: Index compilation and spatial characteristics. China Econ. Q..

[61] Guo D, Zhou L, Chen L (2022). Influence of digital economy on industrial upgrading and employment adjustment. Chin. J. Popul. Sci..

[62] De Falco S (2019). Are smart cities global cities? A European perspective. Eur. Plan. Stud..

[63] Chenhong X, Guofang Z (2022). The spatiotemporal evolution pattern of urban resilience in the Yangtze river delta urban agglomeration based on Topsis-Pso-Elm. Sustain. Cities Soc..

[64] Du Z, Zhang H, Ye Y, Jin L, Xu Q (2019). Urban shrinkage and growth: Measurement and determinants of economic resilience in the Pearl River Delta. J. Geogr. Sci..

[65] Zhou Q, Qiao Y, Zhang H, Zhou S (2022). How does college scale affect urban resilience? Spatiotemporal evidence from China. Sustain. Cities Soc..

[66] Wang K, Ma H, Fang C (2023). The relationship evolution between urbanization and urban ecological resilience in the northern slope economic Belt of Tianshan Mountains, China. Sustain Cities Soc..

[67] Umar M, Ji X, Kirikkaleli D, Xu Q (2020). Cop21 roadmap: Do innovation, financial development, and transportation infrastructure matter for environmental sustainability in China?. J. Environ. Manag..

[68] Machado JAF, Santos Silva JMC (2019). Quantiles via moments. J Econom..

[69] Pace RK, LeSage JP (2009). A sampling approach to estimate the log determinant used in spatial likelihood problems. J. Geogr. Syst..

[70] Agboola OP, Tunay M (2023). Urban resilience in the digital age: The influence of information-communication technology for sustainability. J. Clean Prod..

[71] Chatti W, Majeed MT (2022). Information communication technology (ICT), smart urbanization, and environmental quality: Evidence from a panel of developing and developed economies. J. Clean Prod..

[72] Hao X, Wen S, Xue Y, Wu H, Hao Y (2023). How to improve environment, resources and economic efficiency in the digital era?. Resour. Policy..

[73] Jiang Z, Zhang X, Zhao Y, Li C, Wang Z (2023). The impact of urban digital transformation on resource sustainability: Evidence from a quasi-natural experiment in China. Resour. Policy..

