# Supplementary material for: Digital economy development boosts urban resilience—evidence from China
Source: Sci Rep. 2024 Feb 5;14:2925. doi: 10.1038/s41598-024-52191-4 (PMC11224227; doi:10.1038/s41598-024-52191-4)
Supplement: Supplementary file 1 — Supplementary Information. [file 41598_2024_52191_MOESM1_ESM.pdf]

Digital economy development boosts urban resilience - evidence from China

Haohui Wang[1*], Gang Peng[1], Hongmei Du[2*]

[1] School of Statistics, Southwestern University of Finance and Economic, Chengdu, Sichuan Province, China.

[2] School of Public Administration, China University of Geosciences, Wuhan, Wuhan City, Hubei Province, China.

[*] Corresponding authors:

E-mail: 120020208002@smail.swufe.edu.cn (H.W.)

E-mail: 1202221411@cug.edu.cn (H.D.)

Supplementary Information

1. Data normality test

Before applying MMQR, Jarque and Bera normality analysis was first used to confirm the regularity of the data in this study[1]. As shown in Supplementary Table 1, the results indicate that all variables are non-normally distributed and require a research method that uses non-normally distributed data.

**Variables**	**Ur**	**Dig**	**Lfd**	**Afc**	**Ei**	**Uz**
Jarque-Bera	43000.00	440000.00	68000.00	2162.00	16.42	2594.00
p-value	<0.001	<0.001	<0.001	<0.001	<0.001	<0.001

Supplementary Table 1. Jarque and Bera normality analysis.

2. Cointegration analysis

This paper conducts additional testing using Pedroni's cointegration test. The results of the cointegration test are shown in Supplementary Table 2, and the high significance of the findings confirms the stable long-term relationship between Ur, Dig, Lfd, Afc, Ei, and Uz.

	**Statistic**	**p-value**
Modified Phillips-Perron t	22.553	0.000
Phillips-Perron t	-40.517	0.000
Augmented Dickey-Fuller t	-35.927	0.000

Supplementary Table 2. cointegration analysis.

3. Cross-sectional dependence analysis and slope heterogeneity analysis

To further ensure the accuracy of the findings, both cross-sectional dependence and slope heterogeneity of the data were examined in this paper. The results are shown in Supplementary Tables 3 and 4. The results indicate that there is some

cross-sectional dependence and slope heterogeneity among the variables.

Variables	Ur	Dig	Lfd	Afc	Ei	Uz
Stat	258.62***	525.69***	392.83***	17.45***	103.56***	109.38***
Abs(Corr)	0.584	0.939	0.732	0.492	0.447	0.793

Supplementary Table 3. CD Analysis.

	Ur Dig Lfd Afc Ei Uz
	**Stat**
Delta tilde(Δ_H)	3.338***
Delta tilde Adjusted(Δ_AH)	6.094***

Supplementary Table 4. Slope heterogeneity analysis.

4. Granger causality test

The MMQR model has identified the impact of the digital economy on urban resilience, but the causal relationship between variables still needs to be tested. The Granger causality analysis can effectively deal with the heterogeneity and cross-sectional dependence of the panel data. This paper further analyzes the results using the Dumitrescu and Hurlin causality test[2]. The results, as shown in Supplementary Table 5, further demonstrate the contribution of the digital economy to urban resilience.

Direction	W-bar	Z-bar	p-value
Dig→Ur	3.069	23.181	0.0000
Lfd→Ur	2.512	16.934	0.0000
Afc→Ur	1.937	10.500	0.0000
Ei→Ur	2.098	12.302	0.0000
Uz→Ur	2.944	21.777	0.0000

Supplementary Table 5. Causality analysis.

5. full sample threshold effect test and threshold value test

Threshold variable	Model	F-statistic	p-value	Threshold value		
				10%	5%	1%
Dig	Single Threshold	122.94	0.000	27.009	31.741	40.966
	Double Threshold	58.92	0.053	29.478	62.622	86.447
	Triple Threshold	22.37	0.323	48.518	63.643	99.254

Supplementary Table 6. Test for digital threshold effects.

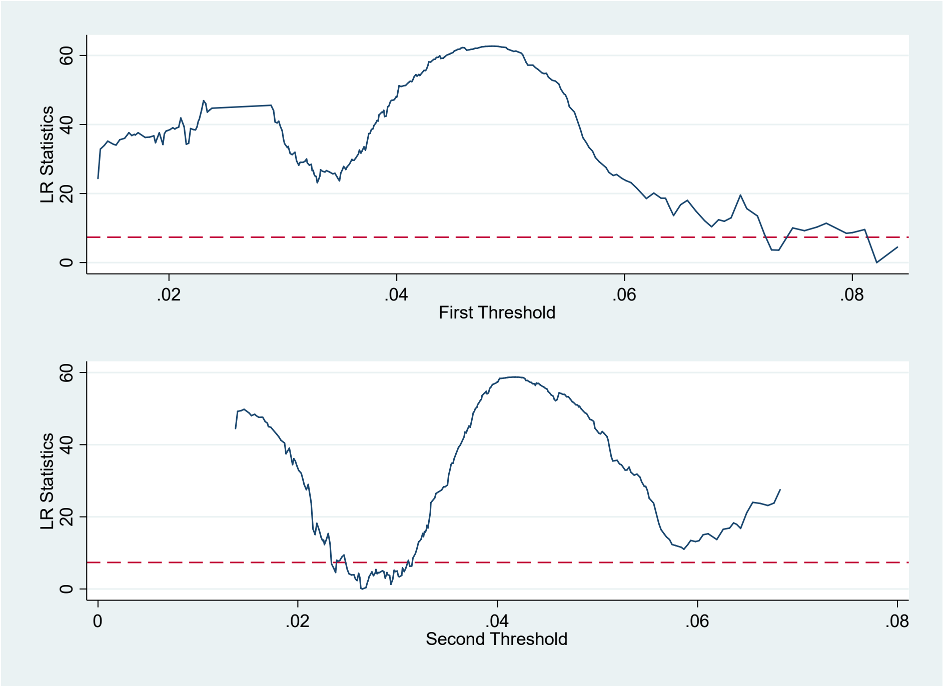

Supplementary Figure 1. Full sample threshold test.

6. spatial correlation test

As shown in Supplementary Table 7, the Moran's I indices for both digital economy and urban resilience are positive, and the corresponding Moran's I indices are significant at the 5% level in most years.

	Ur		Dig	
year	w1	w2	w1	w2

	Moran's I	Z-statistic	Moran's I	Z-statistic	Moran's I	Z-statistic	Moran's I	Z-statistic
2011	0.041^{***}	7.905	0.137^{***}	7.020	0.014^{***}	3.941	0.063^{***}	4.074
2012	0.040^{***}	7.744	0.133^{***}	6.831	0.011^{***}	3.171	0.054^{***}	3.465
2013	0.049^{***}	9.417	0.166^{***}	8.565	0.007^{**}	2.174	0.038^{**}	2.355
2014	0.036^{***}	6.998	0.124^{***}	6.402	0.008^{**}	2.355	0.040^{***}	2.567
2015	0.040^{***}	7.688	0.135^{***}	6.952	0.005^{*}	1.913	0.032^{**}	2.133
2016	0.034^{***}	6.770	0.122^{***}	6.321	0.004^{*}	1.719	0.030^{**}	2.002
2017	0.033^{***}	6.502	0.121^{***}	6.254	0.006^{**}	2.051	0.036^{**}	2.338
2018	0.027^{***}	5.516	0.105^{***}	5.504	0.006^{**}	2.021	0.035^{**}	2.192
2019	0.028^{***}	5.677	0.110^{***}	5.767	0.006^{*}	1.875	0.032^{**}	1.992
2020	0.028^{***}	5.775	0.113^{***}	5.898	0.006^{**}	1.980	0.034^{**}	2.068

Supplementary Table 7. Spatial correlation test.

7. Heterogeneity - rules of urban division

First, Caijing First ranks Chinese cities by five aspects: business resources, transportation hubs, resident activity, lifestyle, and future plasticity. The higher the rank, the higher the overall endowment of the city. From the perspective of city class, first-tier cities, new first-tier cities, second-tier cities, and third-tier cities are divided into high-class city samples, and the remaining cities are divided into low-class city samples.

High-Level Cities	Specific Cities
First-Tier Cities	Shanghai, Beijing, Guangzhou, Shenzhen.
New First-Tier Cities	Chengdu, Chongqing, Hangzhou, Xi'an, Wuhan, Suzhou, Zhengzhou, Nanjing, Tianjin, Changsha, Dongguan, Ningbo, Foshan, Hefei, Qingdao.
Second-Tier Cities	Kunming, Shenyang, Jinan, Wuxi, Xiamen, Fuzhou,

	Wenzhou, Jinhua, Harbin, Dalian, Guiyang, Nanning, Quanzhou, Shijiazhuang, Changchun, Nanchang, Huizhou, Changzhou, Jiaxing, Xuzhou, Nantong, Taiyuan, Baoding, Zhuhai, Zhongshan, Lanzhou, Linyi, Weifang, Yantai, Shaoxing.
Third-Tier Cities	Taizhou, Haikou, Urumqi, Luoyang, Langfang, Shantou, Huzhou, Xianyang, Yancheng, Jining, Hohhot, Yangzhou, Ganzhou, Fuyang, Tangshan, Zhenjiang, Handan, Yinchuan, Nanyang, Guilin, Taizhou, Zunyi, Jiangmen, Jieyang, Wuhu, Shangqiu, Lianyungang, Xinxiang, Huaian, Zibo, Mianyang, Heze, Zhangzhou, Zhoukou, Cangzhou, Xinyang, Hengyang, Zhanjiang, Sanya, Shangrao, Xingtai, Putian Liuzhou, Suqian, Jiujiang, Xiangyang, Zhumadian, Yichang, Yueyang, Zhaoqing, Chuzhou, Weihai, Dezhou, Tai'an, Anyang, Jingzhou, Yuncheng, Anqing, Chaozhou, Qingyuan, Kaifeng, Suzhou, Zhuzhou, Bengbu, Ningde, Lu'an, Yichun, Liaocheng, Weinan, Xuchang.

Supplementary Table 8. Sample list of high-level cities.

Secondly, according to the Chinese government's specific classification of urban agglomerations in each region of China, cities in the Beijing-Tianjin-Hebei Delta, the Yangtze River Delta, the Pearl River Delta, the middle reaches of the Yangtze River, the Central Plains city cluster, and the Chengdu-Chongqing city cluster are divided into key city samples, and other cities are non-key city samples.

Key City Clusters	Specific Cities
Beijing-Tianjin-Hebei Delta	Beijing, Tianjin, Tangshan, Langfang, Baoding, Shijiazhuang, Qinhuangdao, Cangzhou, Chengde, Zhangjiakou, Hengshui,

	Handan, Xingtai.
Yangtze River Delta	Shanghai, Nanjing, Wuxi, Changzhou, Suzhou, Nantong, Yancheng, Yangzhou, Zhenjiang, Taizhou, Hangzhou, Ningbo, Jiaxing, Wenzhou, Huzhou, Shaoxing, Jinhua, Zhoushan, Taizhou, Hefei, Wuhu, Ma'anshan, Tongling, Anqing, Chuzhou, Chizhou, Xuancheng.
City Cluster in the Middle Reaches of the Yangtze River	Wuhan, Huangshi, Ezhou, Huanggang, Xiaogan, Xianning, Xiantao, Qianjiang, Tianmen, Xiangyang, Yichang, Jingzhou, Jingmen, Changsha, Zhuzhou, Xiangtan, Yueyang, Yiyang, Changde, Hengyang, Loudi, Nanchang, Jiujiang, Jingdezhen, Yingtan, Xinyu, Yichun, Pingxiang, Shangrao, Ji'an, Fuzhou.
Pearl River Delta	Guangzhou, Shenzhen, Zhuhai, Foshan, Dongguan, Zhongshan, Jiangmen, Zhaoqing, Huizhou.
Chengdu-Chongqing City Cluster	Chongqing, Chengdu, Zigong, Luzhou, Deyang, Mianyang, Suining, Neijiang, Leshan, Nanchong, Meishan, Yibin, Guang'an, Dazhou, Ya'an, Ziyang.
Central Plains City Cluster	Zhengzhou, Luoyang, Kaifeng, Nanyang, Anyang, Shangqiu, Xinxiang, Pingdingshan, Xuchang, Jiaozuo, Zhoukou, Xinyang, Zhumadian, Hebi, Puyang, Luohe, Sanmenxia, Jiyuan, Changzhi, Jincheng, Yuncheng, Liaocheng, Heze, Suzhou, Huaibei, Fuyang, Bozhou, Bengbu.

Supplementary Table 9. Sample list of key cities.

Third, according to the National Bureau of Statistics of China (NBS) "Statistical System and Classification Standard (17)" China's economic zones division, Chinese cities are categorized into East, West, Central and Northeast.

Economic Zone	Specific Provinces
Eastern Region	Beijing, Tianjin, Hebei, Shanghai, Jiangsu, Zhejiang, Fujian, Shandong, Guangdong, Hainan.
Central Region	Shanxi, Anhui, Jiangxi, Henan, Hubei, Hunan.
Western Region	Inner Mongolia, Guangxi, Chongqing, Sichuan, Guizhou, Yunnan, Tibet, Shaanxi, Gansu, Qinghai, Ningxia, Xinjiang.
Northeast Region	Liaoning, Jilin, Heilongjiang.

Supplementary Table 10. Sample list of regional divisions.

8. heterogeneity analysis of nonlinear effects

Supplementary Table 11 shows the results of the threshold effect test; Supplementary Figures 1-4 show the results of the threshold test; and Supplementary Table 12 shows the results of the threshold regression.

The following conclusions can be drawn by combining the above graphs: First, the key city clusters, high-class cities, and east-west cities pass the threshold test, and the dynamic nonlinear spillover effect of digital economy development on the improvement of cities' risk resistance is further verified. Second, non-key cities, low-class cities, the central region, and the northeastern region do not pass the threshold test, indicating that the non-linear spillover effect of the digital economy is more obvious in developed cities.

	**Threshold variable**	**Model**	**F-statistic**	**p-value**	**Threshold value**		
					**10%**	**5%**	**1%**
Key	Dig	Single Threshold	83.58	0.0000	22.5590	28.5062	37.3364
		Double	27.72	0.0467	21.8256	27.0573	38.9654

		Threshold					
		Triple Threshold	14.06	0.5770	31.9342	36.7409	48.1212
High Level	Dig	Single Threshold	95.24	0.0100	23.2761	25.8431	34.5477
		Double Threshold	19.65	0.1400	21.2804	23.9951	31.2928
		Triple Threshold	10.71	0.2700	16.2489	18.6417	26.6540
East	Dig	Single Threshold	34.43	0.0133	21.6176	27.4288	36.6627
		Double Threshold	19.69	0.1200	20.6175	23.1431	36.4356
		Triple Threshold	9.39	0.4400	17.3919	20.9597	27.5350
West	Dig	Single Threshold	48.63	0.0033	20.8514	26.4749	40.3128
		Double Threshold	17.89	0.0333	13.9481	15.9715	26.3017
		Triple Threshold	5.36	0.6500	13.3728	15.7265	29.3534

Supplementary Table 11. Threshold effect test.

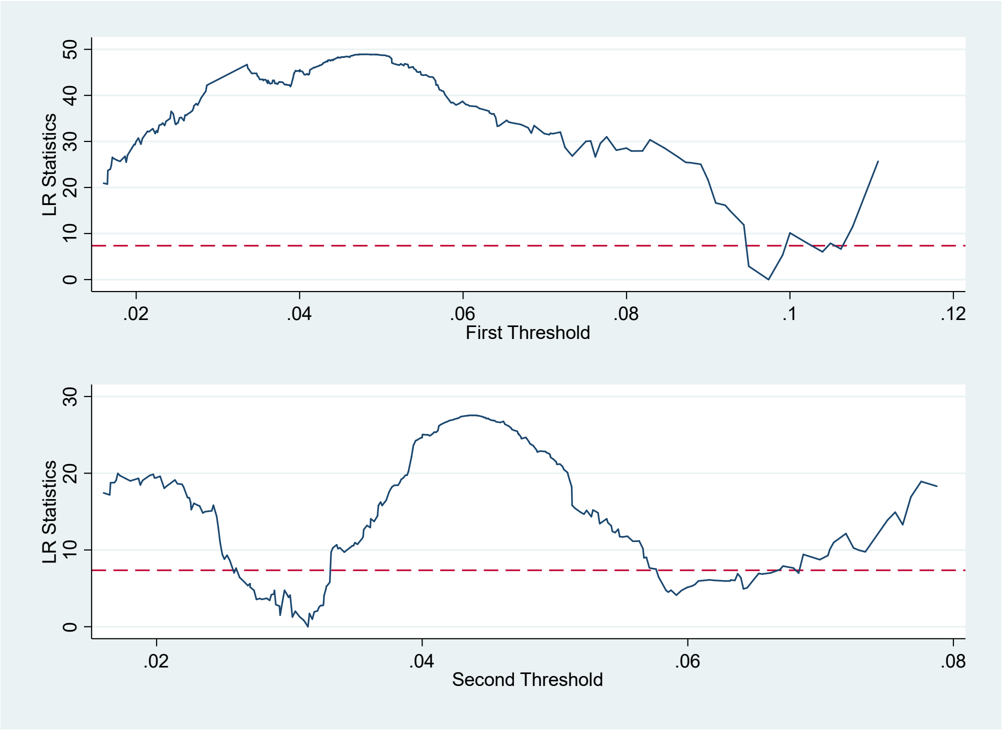

Supplementary Figure 2. Threshold test for key urban clusters.

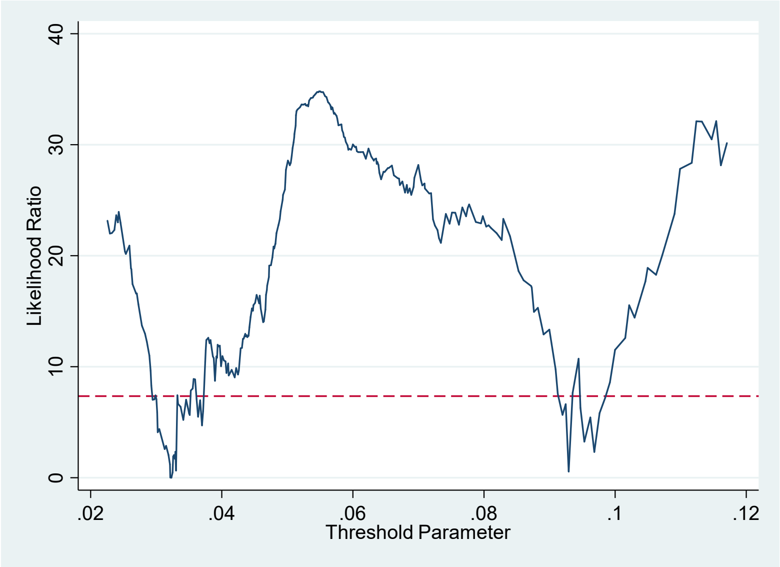

Supplementary Figure 3. High-level city threshold test.

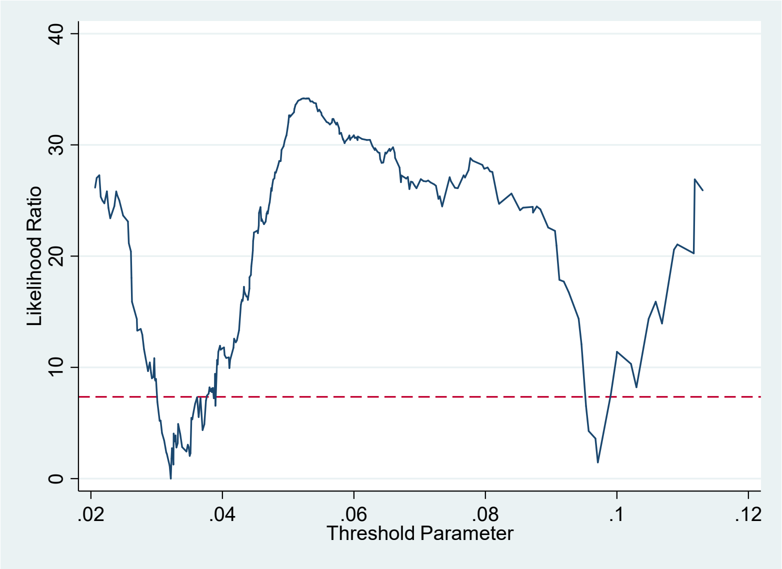

Supplementary Figure 4. Eastern region threshold test.

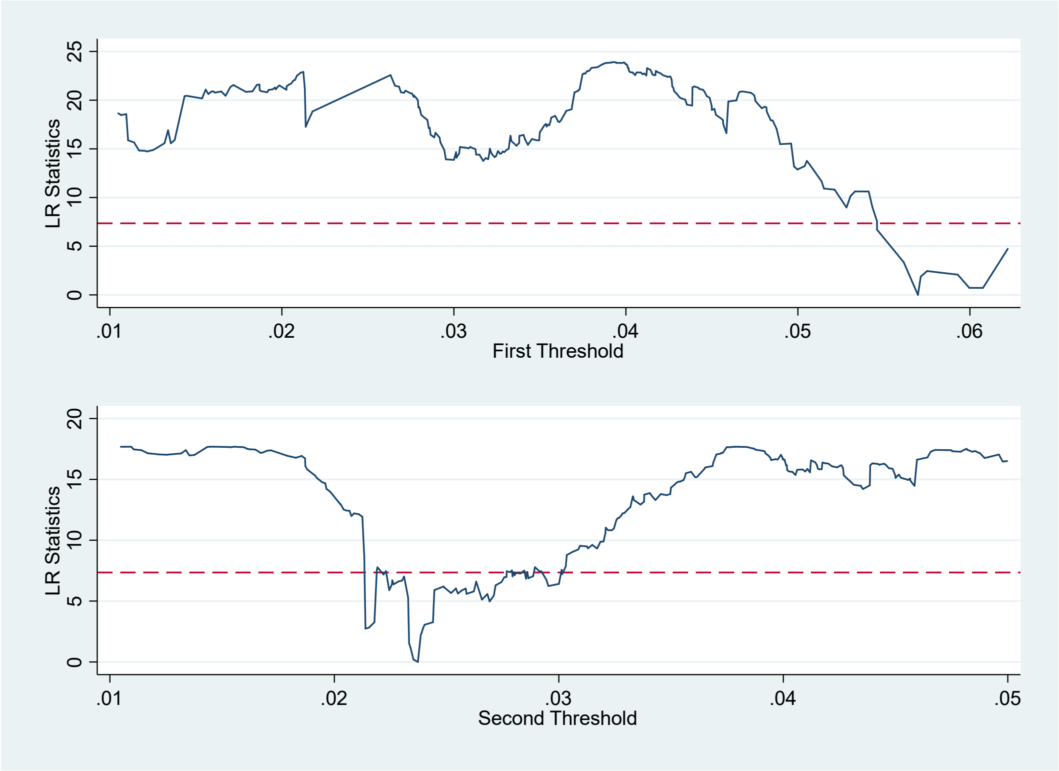

Supplementary Figure 5. Western region threshold test.

Key		**Ur(1)**	**Ur(2)**	**Ur(3)**
	Threshold value	Dig≤0.0314	0.0314<Dig≤0.0974	Dig>0.0974
	Dig	0.891***	0.539***	0.794***

		(0.097)	(0.047)	(0.026)
High Level		Ur(1)		Ur(2)
	Threshold value	Dig≤0.3334		Dig＞0.3334
	Dig	1.234*** (0.093)		0.755*** (0.028)
East	Threshold value	Ur(1)		Ur(2)
	Dig	1.311*** (0.101)		0.798*** (0.034)
		Ur(1)	Ur(2)	Ur(3)
West	Threshold value	Dig≤0.0237	0.0237<Dig≤0.0570	Dig＞0.0570
	Dig	0.841*** (0.159)	0.378*** (0.072)	0.659*** (0.041)

Supplementary Table 12. Regression results of digital threshold model.

References

1. Jarque, C.M., Bera, A.K. A test for normality of observations and regression residuals. *Int. Stat. Rev*. **55**, 163(1987).
2. Dumitrescu, E. & Hurlin, C. Testing for granger non-causality in heterogeneous panels. *Economic Modelling*. **29**, 1450-1460 (2012).
